# Clinical, radiological and laboratory features and outcomes of diffuse alveolar hemorrhage

**DOI:** 10.1038/s41598-026-41864-x

**Published:** 2026-03-06

**Authors:** Juan Ricardo Lutz, Eddy Martínez-Brocato, Sonia Vanessa Segura-Martínez, Gabriela Valentina Baracaldo Amado

**Affiliations:** 1 Pulmonology Department, Hospital Universitario Mayor-Méderi, Bogotá, Colombia; 2 Pulmonology Department, Hospital Universitario San Ignacio, Bogotá, Colombia; 3 Internal Medicine Department, Hospital Universitario San Ignacio, Bogotá, Colombia; 4 Department of Geriatric Medicine, Hospital Universitario San Ignacio, Bogotá, Colombia

**Keywords:** Diffuse alveolar hemorrhage, Bronchoalveolar lavage, Autoimmune disease, Hypoxemia, Diseases, Medical research, Risk factors

## Abstract

Diffuse alveolar hemorrhage (DAH) is a rare and potentially life-threatening syndrome with heterogeneous etiologies and limited regional data from Latin America. We conducted a retrospective cohort study of adults with bronchoalveolar lavage–confirmed DAH managed at two tertiary referral centers in Bogotá, Colombia, between 2019 and 2024. Clinical, radiological, laboratory characteristics, treatments and in-hospital outcomes were analyzed, and factors associated with mortality were explored using logistic regression models. A total of 95 patients were included, with a mean age of 49 ± 18 years; 51% were male. Autoimmune diseases were the most frequent etiology (80%), and ground-glass opacities were observed in 94% of chest computed tomography scans. Immunosuppressive therapy was administered to most patients. Overall, in-hospital mortality was 12%. In univariable analyses, hypoxemia, chest pain, microbiological isolation and use of blood products were associated with mortality, whereas autoimmune etiology was associated with lower risk of death. In multivariable analysis, hypoxemia at admission remained independently associated with in-hospital mortality. These findings highlight the predominance of autoimmune causes of DAH and underscore the importance of early etiological identification and assessment of respiratory severity to improve clinical outcomes.

## Introduction

The earliest description of diffuse alveolar hemorrhage (DAH) is attributed to Virchow, who in the nineteenth century reported what is now considered the first documented case, likely to represent pulmonary hemosiderosis^1^. DAH is a rare yet potentially life-threatening syndrome, with reported in-hospital mortality ranging from 20 to 100%^2,3^. It is defined by a combination of signs and symptoms resulting from intra-alveolar bleeding arising from the pulmonary microcirculation^2,4^. Clinically, it usually presents with dyspnea, hemoptysis, anemia, bilateral pulmonary infiltrates and hypoxemic respiratory failure^5^.

Although its true incidence in the general population is unknown, DAH occurs more frequently in autoimmune conditions. Anti-glomerular basement membrane (anti-GBM) disease is particularly notable, with up to 60% of patients developing DAH^6^. Prevalence in antineutrophil cytoplasmic antibody (ANCA) associated vasculitides is variable, occurring in approximately 50% of granulomatosis with polyangiitis (GPA), 26% of microscopic polyangiitis (MPA) and 10% of eosinophilic granulomatosis with polyangiitis (EGPA) cases^7^. On histopathological examination, these forms are commonly characterized by pulmonary capillaritis with a perivascular neutrophilic infiltrate^8^. In contrast, hemorrhage without underlying vascular inflammation or destruction is termed “bland hemorrhage,” and may result from elevated left ventricular filling pressures, coagulopathies, medications or other systemic conditions^2,9–11^.

Bronchoalveolar lavage (BAL) remains a fundamental diagnostic tool in DAH, allowing early and reliable confirmation^2,12,13^. A progressively bloodier return across sequential aliquots during BAL is a characteristic finding^14^, and the presence of ≥ 20% hemosiderin—laden macrophages in BAL fluid is considered a key diagnostic threshold^15^.

Management focuses on two pillars: supportive care—addressing respiratory failure, hemodynamic instability and metabolic disturbances—and treatment of the underlying cause^9^.

Given the limited information available in Colombia, largely restricted to isolated case reports or small series, this study aims to provide a detailed description of the clinical, radiological and laboratory features of DAH in two fourth-level hospitals in Bogotá, and to explore differences between autoimmune and non-autoimmune forms as well as factors associated with in-hospital mortality.

## Methods

A descriptive, retrospective study with an analytical component was conducted in two fourth-level hospitals in Bogotá, Colombia (Hospital Universitario San Ignacio and Hospital Universitario Mayor–Méderi between 2019 and 2024. The study was designed and reported in accordance with the Strengthening the Reporting of Observational Studies in Epidemiology (STROBE) guidelines. We included patients aged 18 years or older with a diagnosis of DAH, defined by compatible clinical and radiological findings together with a BAL cytology showing more than 20% hemosiderin—laden macrophages. Patients in whom data could not be retrieved or whose medical records were incomplete were excluded.

The study protocol was approved by the ethics committees of both institutions (reference numbers FM-CIE-1410-24 and CEISH-2025086), and the study was conducted in accordance with the principles of the Declaration of Helsinki. As this was an observational design without direct interventions, informed consent was not required.

**Fig. 1:**
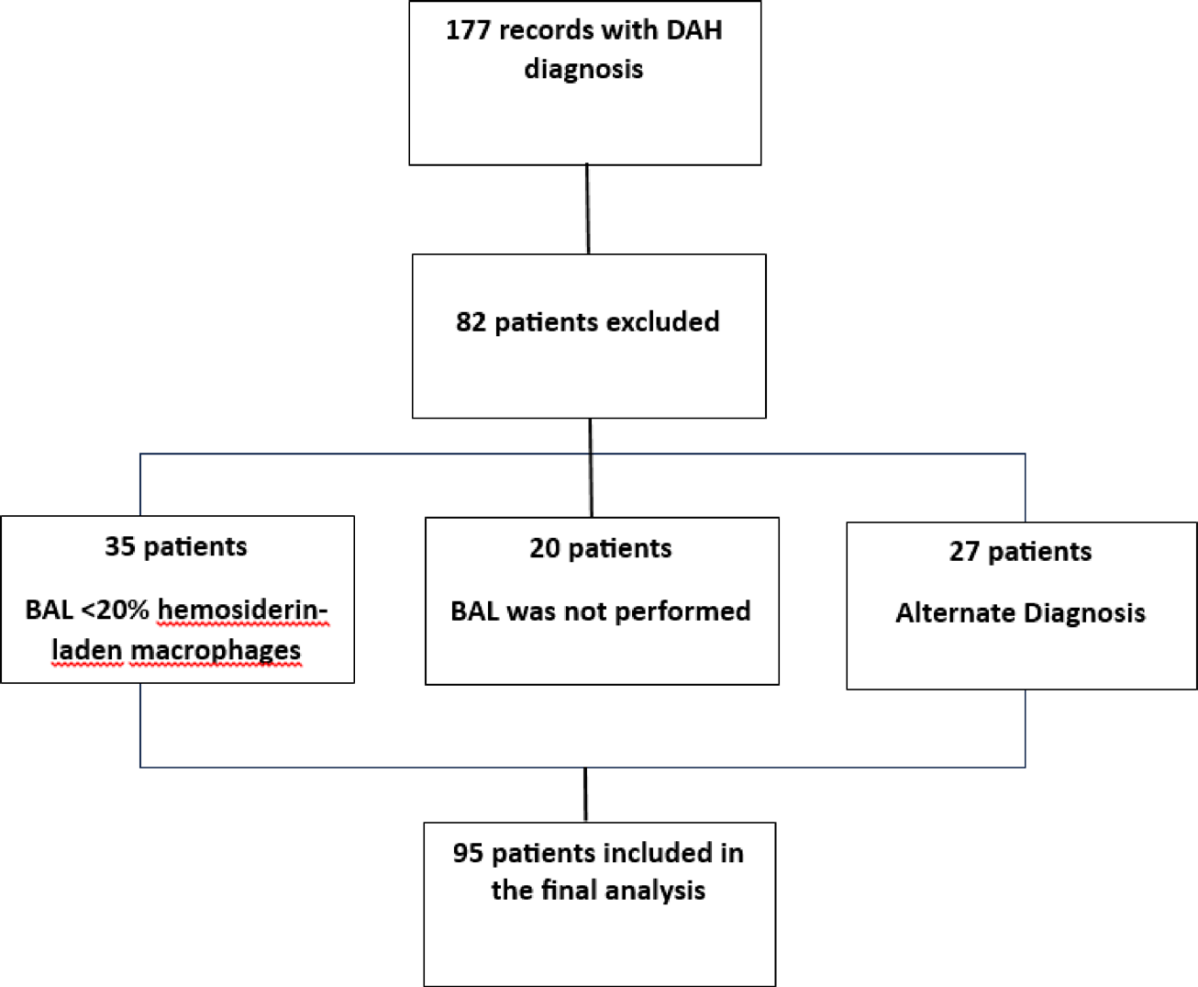
Flowchart of clinical records review.

The sample size was determined by the total number of eligible cases identified during the study period. Information was collected through review of electronic medical records, from which demographic, clinical, radiological, laboratory, treatment and etiological variables, as well as outcomes, were extracted. The primary outcome was in-hospital mortality. A total of 183 medical records were screened; 35 were excluded because BAL showed fewer than 20% hemosiderin—laden macrophages, 20 patients had no BAL performed, and 27 had an alternative diagnosis, resulting in 95 patients included in the final analysis. Figure 1 presents the identification and exclusion process.

Group comparisons were performed between autoimmune-related and non-autoimmune/indeterminate DAH. In addition, a multivariable logistic regression model was constructed to explore factors associated with mortality. Model discrimination was assessed using the area under the ROC curve, and calibration was evaluated through corresponding tests and graphical inspection. Analyses were conducted using complete cases; no imputation methods were applied.

Given the retrospective design, the study is subject to selection bias, as only patients with available BAL results were included, potentially excluding individuals in whom the procedure was not performed due to clinical instability or similar considerations. Efforts were made to minimize information bias through standardized extraction of clinical and laboratory data, although variability in record quality may have contributed to misclassification.

All analyses were performed using R software version 4.5.0 (R Foundation for Statistical Computing, Vienna, Austria). Variables were initially examined for completeness, internal consistency and distribution. Categorical variables were summarized as frequencies and percentages; quantitative variables were described using mean and standard deviation (SD) or median and interquartile range (IQR), according to distribution assessed with the Shapiro–Wilk test.

Group comparisons were made between patients with autoimmune etiology and those with non-autoimmune or indeterminate causes. For bivariate comparisons, Pearson’s chi-square or Fisher’s exact test was used for categorical variables, and Student’s t-test or the Wilcoxon–Mann–Whitney test for continuous variables depending on distribution.

Associations between each independent variable and the primary outcome (in-hospital mortality) were evaluated using univariable binary logistic regression, expressed as odds ratios (ORs) with 95% confidence intervals (95% CIs). Variables with *p* < 0.20 in the univariable analysis, along with those deemed clinically relevant (age, sex, hypoxemia, admission PaO_2_, anemia and creatinine), were entered into the multivariable model. To reduce model overfitting, the number of predictors was limited following the 10 events-per-variable rule.

Model calibration was assessed using the Hosmer–Lemeshow test, and discrimination using the area under the ROC curve (AUC). Collinearity among predictors was evaluated through the variance inflation factor (VIF). Statistical significance was defined as *p* < 0.05, and results were interpreted cautiously given the retrospective, non-randomized nature of the study.

## Results

**Fig. 2:**
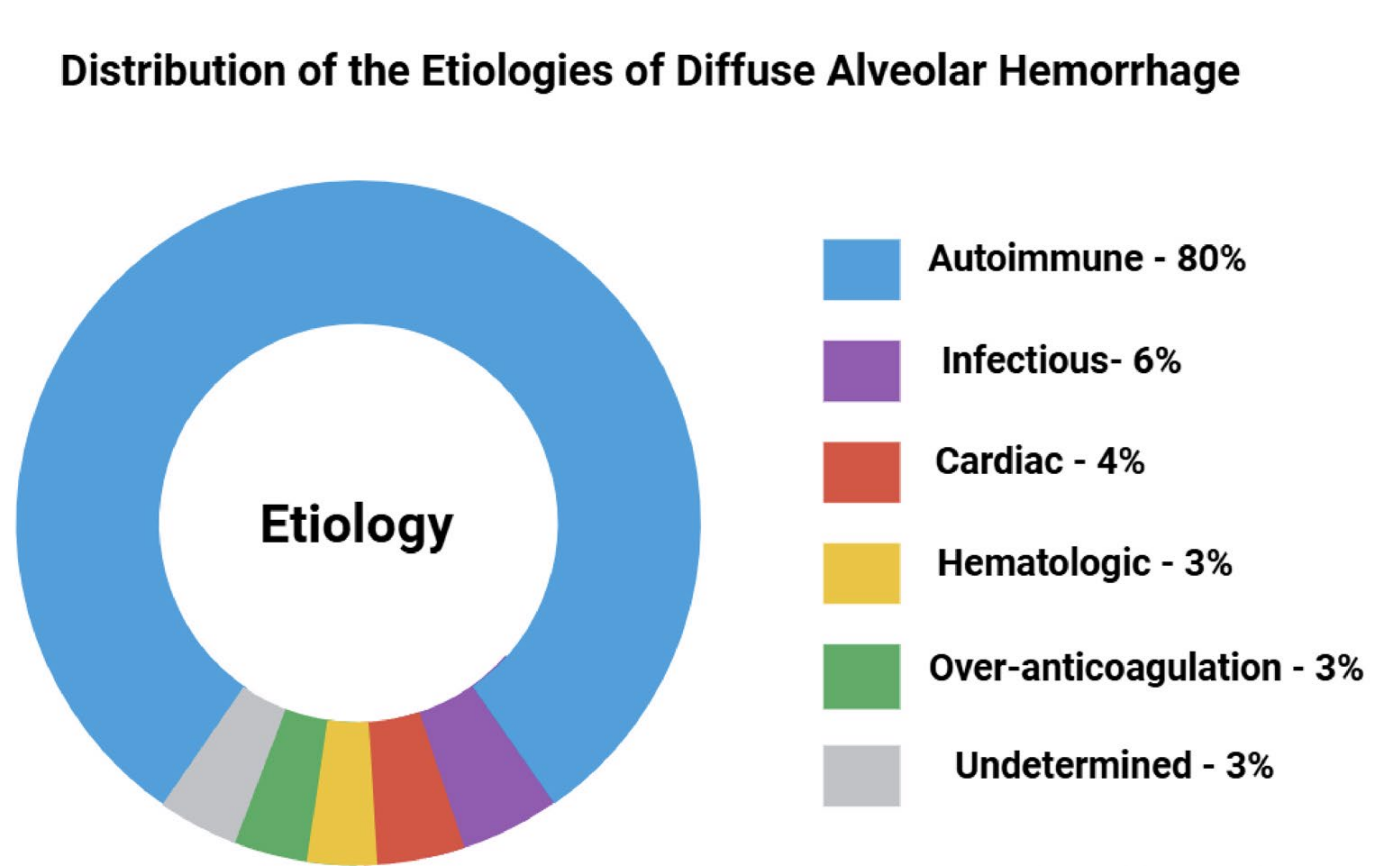
Distribution of the etiologies of DAH.

Throughout the observation window, a total of 95 patients with a diagnosis of DAH were identified (baseline characteristics are presented in Table 1). Of these, 48 (51%) were male, and the mean age at presentation was 49 ± 18 years. The distribution of etiologies is shown in Fig. 2. Autoimmune disease was the most frequent etiology (80%), followed by infectious (6%), cardiac (4%), hematological (3%) and anticoagulation-related causes (3%). In three patients (3%), no identifiable etiology was established. A total of 69 patients (72%) had some degree of immunocompromise, most commonly secondary to immunosuppressive therapy or underlying comorbidities. 18% were on long-term anticoagulation. The most prevalent comorbidities were chronic kidney disease (54%), hypertension (44%) and hypothyroidism (28%). Additionally, 25% of patients had a prior diagnosis of autoimmune disease.

**Table 1:** Baseline characteristics of the study population.

	Autoimmune (N = 76)	Non-autoimmune (N = 19)	*p* Value
Demographic variables			
Sex	M: 36 (47%) F: 40 (53%)	M: 12 (63%) F: 7 (37%)	0.2
Age [IQR]	55 [32, 65]	48 [27, 67]	0.8
Comorbidities			
Hypertension	0 (0%)	1 (5.3%)	0.2
Smoking History	34 (45%)	8 (42%)	0.8
Diabetes	4 (5.3%)	1 (5.3%)	> 0.9
Chronic Kidney Disease	44 (58%)	8 (42%)	0.2
Hypothyroidism	24 (32%)	3 (16%)	0.2
COPD	1 (1.3%)	1 (5.3%)	0.4
Pulmonary Hypertension	0 (0%)	1 (5.3%)	0.2
Heart Failure	6 (7.9%)	6 (32%)	0.013
HIV	0 (0%)	2 (11%)	0.038
Tuberculosis	2 (2.6%)	2 (11%)	0.2
B cell Lymphoma	0 (0%)	4 (21%)	0.001
Myelodysplasic Syndrome	0 (0%)	1 (5.3%)	0.2
Acute Myeloid Leukemia	0 (0%)	1 (5.3%)	0.2
Systemic Lupus Erythematosus	19 (25%)	2 (11%)	0.2
Sjögren´s Syndrome	2 (2.6%)	0 (0%)	> 0.9
Rheumatoid Arthritis	6 (7.9%)	0 (0%)	0.3
Antiphospholipid Syndrome	7 (9.2%)	3 (16%)	0.4
ANCA-associated vasculitis			< 0.001
MPA	23 (30%)	1 (5.3%)	
GPA	16 (21%)	0 (0%)	
EGPA	1 (1.3%)	0 (0%)	
Indeterminate	14 (18%)	0 (0%)	
Immunocompromise	57 (75%)	12 (63%)	0.3
Signs and Symptoms			
Fever	16 (21%)	5 (26%)	0.8
Dyspnea	52 (68%)	11 (58%)	0.4
Cough	41 (54%)	10 (53%)	> 0.9
Hemoptysis	43 (57%)	9 (47%)	0.5
Chest Pain	23 (30%)	4 (21%)	0.4
Hypoxemia	45 (61%)	14 (78%)	0.2
PaO2 on admission	71 [63, 81]	74 [65, 84]	0.6
Anemia	61 (80%)	14 (74%)	0.5
Hemoglobin decline	30 (39%)	8 (42%)	0.8
Use of Warfarin	5 (6.6%)	5 (26%)	0.025
Use of DOACs	2 (2.6%)	1 (5.3%)	0.5
Use of LMWH	3 (3.9%)	1 (5.3%)	> 0.9
Laboratory findings			
Hemoglobin (g/dl) [IQR]	9.4 [7.5, 12.0]	9.9 [8.5, 13.0]	0.5
Platalet count [IQR]	273,000 [208,500,384,000]	242,000 [54,000,302,000]	0.052
Creatinine (mg/dl) [IQR]	3.20 [1.28, 5.40]	1.63 [0.71, 4.32]	0.068
BUN (mg/dl) [IQR]	41 [26, 62]	32 [14, 51]	0.13
INR [IQR]	1.10 [1.04, 1.28]	1.17 [1.05, 1.60]	0.2
ESR (mm/hr) [IQR]	44 [22, 76]	28 [13, 74]	0.4
ANA positive	21 (28%)	1 (5.3%)	0.082
ANCA positive	29 (38%)	1 (5.3%)	0.009
Anti-SS-A positive	3 (3.9%)	1 (5.3%)	0.15
Anti-SS-B positive	1 (1.3%)	1 (5.3%)	0.12
Anti-MPO positive	10 (13%)	1 (5.3%)	0.3
Anti-PR3 positive	12 (16%)	0 (0%)	0.044
Low C3 Level	12 (16%)	1 (5.3%)	0.3
Low C4 Level	18 (24%)	1 (5.3%)	0.11
Microbiological findings	12 (16%)	8 (42%)	0.012
*K. pneumoniae*	5 (6.8%)	3 (16%)	
*M. catarrhalis*	1 (1.4%)	0 (0%)	
*S. pneumoniae*	0 (0%)	1 (5.3%)	
*S. aureus*	1 (1.4%)	1 (5.3%)	
*S. agalactiae*	1 (1.4%)	0 (0%)	
*S. hominis*	0 (0%)	1 (5.3%)	
*C. albicans*	2 (2.7%)	0 (0%)	
*C. glabrata*	1 (1.4%)	0 (0%)	
*SARS-CoV2*	0 (0%)	1 (5.3%)	
*M. tuberculosis*	1 (1.4%)	0 (0%)	
*Aspergillus spp*	0 (0%)	1 (5.3%)	
Imaging findings (on CT)			
Ground-glass opacities	73 (96%)	16 (84%)	0.092
Consolidations	9 (12%)	6 (32%)	0.071
Etiology			< 0.001
Autoimmune	76 (100%)	0 (0%)	
Infectious	0 (0%)	4 (21%)	
Cardiac	0 (0%)	6 (32%)	
Hematologic	0 (0%)	3 (16%)	
Over-anticoagulation	0 (0%)	3 (16%)	
Undetermined	0 (0%)	3 (16%)	
Treatment			
Steroids	75 (99%)	5 (26%)	< 0.001
Cyclophosphamide	49 (64%)	0 (0%)	< 0.001
Plasma Exchange	36 (47%)	0 (0%)	< 0.001
Rituximab	16 (21%)	0 (0%)	0.036
Diuretics	10 (13%)	3 (16%)	0.7
Antibiotics	10 (13%)	8 (42%)	0.008
Hemodialysis	19 (25%)	4 (21%)	> 0.9
Use of Blood Products	23 (30%)	6 (32%)	> 0.9
Anticoagulation Adjustment	1 (1.3%)	4 (21%)	0.005
Heart Transplant	0 (0%)	1 (5.3%)	0.2
Outcomes			
Relapse	14 (18%)	1 (5.3%)	0.3
Death	7 (9.2%)	4 (21%)	0.2

ANCA, Antineutrophil Cytoplasmic Antibody; ANA, Antinuclear Antibodies; Anti-MPO, Anti-Myeloperoxidase; Anti-PR3, Anti-Proteinase 3; Anti-SS-A, Anti-Sjögren’s Syndrome-related antigen A; Anti-SS-B, Anti-Sjögren’s Syndrome-related antigen B; BUN, Blood Urea Nitrogen; C3/C4, Complement component 3/4; COPD, Chronic Obstructive Pulmonary Disease; CT, Computed Tomography; DAH, Diffuse Alveolar Hemorrhage; DOACs, Direct Oral Anticoagulants; EGPA, Eosinophilic Granulomatosis with Polyangiitis; ESR, Erythrocyte Sedimentation Rate; F, Female; GPA, Granulomatosis with Polyangiitis; HIV, Human Immunodeficiency Virus; INR, International Normalized Ratio; IQR, Interquartile Range; LMWH, Low Molecular Weight Heparin; M, Male; MPA, Microscopic Polyangiitis; PaO_2_, Partial pressure of oxygen in arterial blood; SLE, Systemic Lupus Erythematosus.

Most patients presented with dyspnea (66%), hypoxemia (62%) and hemoptysis (55%). Anemia was frequent (78%); 38 patients (40%) exhibited a significant drop in hemoglobin and 29 (30%) required administration of blood components.

The mean percentage of hemosiderin—laden macrophages in BAL fluid was 50 ± 26%. Positive cultures were obtained in 20 patients (21%), with *Klebsiella pneumoniae* being the most identified pathogen. On chest CT, ground-glass opacities were the most frequent finding (94%), with concomitant consolidation observed in 16% of cases, while isolated consolidation was present in only 6%.

Treatment was guided by the underlying cause of DAH. Systemic corticosteroids were administered to 84% of patients, often in combination with cyclophosphamide (52%), plasma exchange (38%) or rituximab (17%). Hemodialysis was required in 23 patients (24%), antibiotic therapy in 18 (19%) and adjustment of anticoagulation in 5 (5%). One patient underwent heart transplantation. Sixteen percent had experienced at least one prior episode of DAH. Overall, in-hospital mortality was 12%.

## Discussion

In this retrospective cohort of adults with diffuse alveolar hemorrhage confirmed by bronchoalveolar lavage, autoimmune disease represented the leading underlying cause, consistent with previous reports in which ANCA-associated vasculitis and other immune-mediated conditions account for most adult cases of DAH. A substantial proportion of patients were immunocompromised, mainly due to immunosuppressive therapy or disease-related factors, highlighting the increased vulnerability of this population to developing DAH, either through reactivation of the underlying autoimmune condition or secondary pulmonary and infectious complications^16^. In contrast, infectious etiologies were considered the primary cause in patients without evidence of an associated connective tissue disease or other identifiable underlying condition, particularly in the presence of unilateral radiological consolidation. Nevertheless, distinguishing whether infection represents a primary driver of DAH or a secondary consequence of lung injury and immunosuppression remains challenging. Infectious causes have been reported in up to 60% of DAH cases^17^, predominantly bacterial, acting as precipitating or contributing factors, especially among immunocompromised patients. In our cohort, decisions regarding antimicrobial therapy were guided by the treating physicians based on overall clinical judgment.

**Fig. 3:**
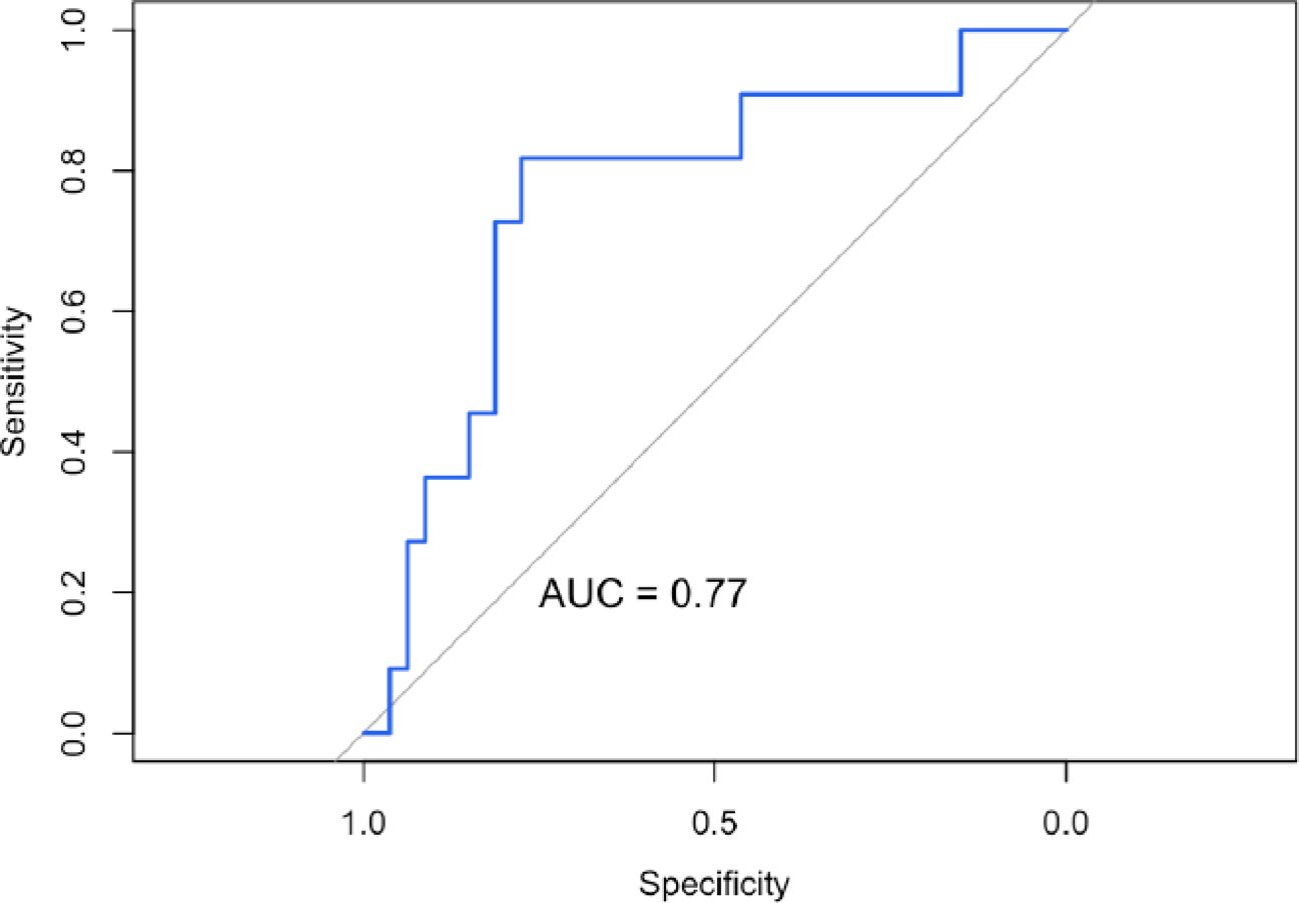
ROC curve showing the discriminatory performance of the model, with an AUC of 0.77.

From a clinical standpoint, the most common presenting signs and symptoms were dyspnea, cough, and hypoxemia, consistent with the well-recognized presentation of acute or subacute respiratory compromise described in previous reports^13^. With respect to imaging findings, bilateral ground-glass opacities were the predominant and most characteristic pattern, observed in 94% of cases; ground-glass opacities with associated consolidation were present in 16%, while isolated consolidation was identified in 6%. This distribution aligns with established radiologic features of DAH and represents one of the most important diagnostic clues in the early phases of the disease^18^. While imaging patterns were not analyzed in a granular comparative fashion, the study emphasizes their diagnostic relevance in real-world clinical practice.

Demographic factors and overall comorbidities were comparable between autoimmune and non-autoimmune or indeterminate groups, the autoimmune subset showed a significantly higher prevalence of ANCA-associated vasculitis (70% vs. 5%), led by microscopic polyangiitis aligning with data observed in similar study populations^7^. Importantly, the isolated presence of ANCA positivity does not necessarily imply an underlying vasculitic process in the absence of compatible clinical, laboratory, and/or histopathological criteria.

Elevated anti-PR3 antibody titters were found in 16% of autoimmune cases, consistent with previously reported studies^19^. Interestingly, our cohort exhibited a higher proportion of patients with reduced C4 complement levels, a finding that contrasts with some of the existing literature^13,20^ and may reflect population-specific differences, local disease characteristics, or variations in immunological profiling.

Recurrent DAH was observed in 16% of patients, highlighting the relapsing course of this condition, particularly among those with autoimmune vasculitis. These findings support the need for sustained immunologic control and long-term follow-up, given that many of our recurrent episodes were linked to discontinuation or inadequate maintenance of immunosuppressive therapy.

Regarding outcomes, univariate analysis identified several variables associated with in-hospital mortality (Table 2), although confidence intervals were wide owing to the limited number of events (n = 11). Autoimmune etiology was associated with lower mortality, a finding consistent with prior evidence highlighting the prognostic value of early diagnosis and timely initiation of immunosuppressive therapy in immune-mediated diseases^21^. The multivariable model (Table 3) demonstrated acceptable discriminative performance (AUC 0.77) (Fig. 3), supporting the potential clinical relevance of the selected predictors—particularly hypoxemia—as prognostic indicators in DAH. Notably, overall mortality in our cohort was lower than that reported in previously published series, which may be partly explained by patient management at quaternary referral centers with access to advanced diagnostic modalities, aggressive medical therapies, and a multidisciplinary care approach.

**Table 2:** Univariate analysis of factors associated with mortality.

Variable	OR (95% CI)
Hypoxemia	6.5 (1.2–36.5)
Chest pain	3.6 (0.99–13.3)
Use of blood products	3.2 (0.9–11.6)
Positive bacterial culture	3.3 (0.9–12.2)
Autoimmune etiology	0.05 (0.00–0.51)

**Table 3:** Multivariate analysis of factors associated with in-hospital mortality.

Variable	OR (95% CI)	*p* Value
Age	0.99 (0.96–1.03)	0.6
Male sex	2.04 (0.53–8.83)	0.3
Hypoxemia	6.23 (1.07–119)	0.092
PaO_2_ at admission	1.01 (0.98–1.04)	0.5
Anemia	1.23 (0.23–9.90)	0.8
Creatinine	0.92 (0.68–1.05)	0.5

No significant associations were observed between mortality and other clinically important variables such as age, sex, or anemia. With respect to renal function, the lack of association between creatinine levels and fatal outcomes is likely explained by the high prevalence of pre-existing chronic kidney disease at presentation, which may have obscured the relationship between acute renal dysfunction and mortality. Pulmonary severity was partially captured through hypoxemia at admission.

One of the main strengths of this study lies in the size of the cohort, positioning it among the largest locally reported series of DAH. Additionally, we performed a comprehensive clinical, radiological, and therapeutic characterization, with diagnostic confirmation obtained through bronchoalveolar lavage and chest computed tomography in all cases. Another notable strength is the analytical assessment of prognosis, which incorporated a multivariable model with estimation of the area under the ROC curve, allowing for an objective exploration of potential predictors of in-hospital mortality.

These findings should be interpreted considering several limitations. First, the retrospective design and the study’s restriction to two fourth-level hospitals limit the generalizability and applicability of our results to diverse healthcare settings. Second, the low number of fatal events decreased the statistical power of the multivariable analysis and widened confidence intervals, thus necessitating cautious interpretation of the associated mortality factors. Third, data regarding symptom onset and exact timing of treatment initiation were not uniformly available and could not be reliably analyzed and the lack of post-discharge follow-up precludes any assessment of long-term mortality and limits the prognostic value of the study. Finally, the immunologic workup was not standardized and was guided by clinical context and physician judgment.

Our study shows that autoimmune-related DAH constitutes the most frequent etiology, with ANCA-associated vasculitis being the most common underlying cause. Early identification of the underlying etiology and timely initiation of intensive immunosuppressive therapy in autoimmune cases were associated with lower in-hospital mortality compared with non-autoimmune forms, underscoring the clinical relevance of recognizing potentially treatable immune-mediated diseases. Furthermore, hypoxemia at admission emerged as an independent clinical marker of poor prognosis, highlighting the importance of the initial degree of respiratory dysfunction as a prognostic indicator. Hypoxemia appeared to be a stronger marker of mortality than PaO₂ in the multivariable analysis, likely because arterial blood gases were frequently obtained while patients were receiving supplemental oxygen.

From a pathophysiological standpoint, hypoxemia amplifies systemic inflammatory cascades and disrupts endothelial–epithelial homeostasis; in the context of lung injury and inflammation, this impaired host response increases susceptibility to secondary infections, including bacterial pneumonia, further worsening clinical outcomes, particularly in patients with acute respiratory distress syndrome (ARDS)^22^.Future studies should explore the role of inflammatory and immunologic biomarkers to improve prognostic stratification in DAH.

These results reinforce the critical importance of a multidisciplinary approach and the prompt identification of the underlying cause as key strategies to optimize the management and improve outcomes for patients with DAH.

## Data Availability

The data that support the findings of this study are available from the corresponding author upon reasonable request, subject to approval from the institutional ethics committees.
